# Dual role of TLR4 in bacterial meningitis through regulating endothelial pyroptosis and inflammatory response during extraintestinal pathogenic *Escherichia coli* infection

**DOI:** 10.3389/fimmu.2025.1581696

**Published:** 2025-08-01

**Authors:** Kaixiang Jia, Yangyang Du, Weixian Lin, Xuefeng Cao, Jialu Zhang, Lianci Peng, Zhiwei Li, Rendong Fang

**Affiliations:** ^1^ Joint International Research Laboratory of Animal Health and Animal Food Safety, College of Veterinary Medicine, Southwest University, Chongqing, China; ^2^ Department of Microbiology and Immunology, Stanford University, Stanford, CA, United States; ^3^ National Center of Technology Innovation for Pigs, Chongqing, China

**Keywords:** bacterial meningitis, toll-like receptor 4, blood-brain barrier, endothelial pyroptosis, host response

## Abstract

Bacterial meningitis is a severe central nervous system infection with incompletely understood pathogenesis. Here, we investigated the role of Toll-like receptor 4 (TLR4) in blood-brain barrier disruption induced by extraintestinal pathogenic *Escherichia coli* (ExPEC). *In vitro* studies revealed that ExPEC infection upregulated TLR4 expression in human brain microvascular endothelial cells and induced pyroptosis and tight junction protein degradation. TLR4 inhibition by TAK-242 significantly reduced pyroptosis and inflammatory responses but exacerbated tight junction disruption and bacterial invasion. In macrophages, TLR4 inhibition similarly attenuated pyroptosis and inflammatory responses. Interestingly, despite enhanced blood-brain barrier disruption and increased bacterial burden, TLR4-deficient mice showed significantly improved survival. Transcriptome analysis revealed that TLR4 deficiency triggered comprehensive reprogramming of host responses, characterized by both suppressed inflammatory damage and enhanced tissue homeostatic processes. This study demonstrates for the first time that endothelial pyroptosis is a novel mechanism for ExPEC-induced blood-brain barrier disruption and reveals the crucial role of TLR4 in balancing protective and destructive host responses, providing new insights for therapeutic strategies against bacterial meningitis.

## Introduction

Bacterial meningitis is a severe infectious disease of the central nervous system (CNS) characterized by high morbidity and mortality rates, drawing significant attention worldwide (1). The disease develops when circulating pathogens penetrate the blood-brain barrier (BBB) and invade the brain, where they multiply and induce the release of pro-inflammatory factors and toxic compounds, ultimately leading to meningitis (2). Various bacteria can cause meningitis, including *Streptococcus suis* (3), S*treptococcus pneumoniae* (4), *Neisseria meningitidis* (1), *Haemophilus influenzae* (5), and *Escherichia coli* (6). However, the mechanisms by which pathogens breach the BBB, a prerequisite for causing meningitis (7), remain incompletely understood.

The BBB is a highly selective barrier that plays a crucial role in maintaining CNS microenvironment homeostasis and facilitating communication with systemic compartments (8). It comprises various brain components, including endothelial cells, pericytes, neurons, microglia, astrocytes, and basement membrane (8). Among these, endothelial cells form a continuous barrier through tight junctions (TJs), adherens junctions (AJs), and gap junctions. TJs, composed of occludin, claudins, junctional adhesion molecules (JAMs), and zonula occludens (ZO) family proteins, are believed to regulate paracellular transport by forming high-resistance barriers (9). Since bacterial entry into the brain and subsequent meningitis development critically depends on breaching the BBB, understanding how bacteria compromise BBB integrity is of great importance.

Toll-like receptor 4 (TLR4), a member of the Toll-like receptor (TLR) family, participates in innate immunity and mediates inflammatory responses through recognition of lipopolysaccharide (LPS) or bacterial endotoxins (10). However, excessive TLR4 activation can disrupt immune homeostasis through the production of pro-inflammatory cytokines and chemokines (11), contributing to the development and progression of various diseases, including not only sepsis (12), but also Alzheimer’s disease (13) and cancer (14). Current research on TLR4 in BBB function has largely focused on damage-associated molecular patterns (DAMP)-induced diseases, with studies showing that TLR4^-/-^ mice exhibit significantly reduced early inflammation and attenuated secondary brain injury following cerebral hemorrhage (15). In pathogen-associated molecular patterns (PAMP)-related research, studies using Japanese encephalitis virus (JEV) infection found that while TLR3-/- mice were highly susceptible to the infection, TLR4^-/-^ mice showed enhanced resistance, displaying mild CNS inflammation characterized by reduced viral load, leukocyte infiltration, and pro-inflammatory cytokine expression (16). However, the role of TLR4 in *E. coli* breaching of the BBB remains unclear.

Pyroptosis is a form of inflammatory programmed cell death that plays important roles in both innate immunity and inflammatory diseases (17). This process is executed by the gasdermin family of pore-forming proteins, including GSDMA, GSDMB, GSDMC, GSDMD, GSDME, and GSDMF (PJVK/DFNB59) (17). Gasdermin-mediated pyroptosis has emerged as a key mechanism in various pathological conditions, as demonstrated by studies showing that the TLR4/TAK1/IRF7 axis promotes Parkinson’s disease progression through NLRP3-dependent pyroptosis (18). Notably, recent research has revealed that BBB breakdown during bacterial infection is primarily mediated by caspase-11 non-canonical inflammasome signaling rather than TLR4-dependent pathways (19). However, whether pyroptosis contributes to extraintestinal pathogenic *E. coli* (ExPEC)-induced BBB disruption remains unknown.

Given these considerations, investigating the role of TLR4 in ExPEC-induced BBB disruption is crucial. In this study, we established an ExPEC-induced meningitis model using human brain microvascular endothelial cells (hCMEC/D3) and TLR4 knockout (TLR4^-/-^) mice to explore the role of TLR4 in endothelial barrier disruption during bacterial meningitis. We also investigated its role in systemic inflammation using mouse peritoneal macrophages. Our findings provide valuable insights for targeting TLR4 in the prevention and treatment of bacterial meningitis and neuroinflammation.

## Materials and methods

### Bacterial strains

The ExPEC strain RS218 was kindly provided by Professor Xiangru Wang (College of Veterinary Medicine, Huazhong Agricultural University). The bacteria were cultured on LB agar plates (Qingdao Hope Bio-Technology Co., Ltd., China) at 37°C for 12 hours. Single colonies were selected and cultured in LB broth (Qingdao Hope Bio-Technology Co., Ltd., China).

### Mice

Wild-type (WT) C57BL/6 mice were purchased from the Chongqing Academy of Chinese Materia Medica (Chongqing, China), while TLR4^-/-^ mice were kindly provided by Professor Feng Shao from the National Institute of Biological Sciences (Beijing, China) and were maintained in our laboratory. All mice were kept under Specific Pathogen-Free (SPF) conditions and used at 8–10 weeks of age. This study was approved by the Institutional Animal Care and Use Committee (IACUC) of Southwest University, Chongqing, China (IACUC-20221022-07).

### Cell culture

The human cerebral microvascular endothelial cells (hCMEC/D3) were purchased from Jennio Biotech (Guangzhou Jennio Biotech Co., Ltd., China). The hCMEC/D3 cells were cultured in Dulbecco’s Modified Eagle Medium (DMEM; Gibco, USA) supplemented with 10% fetal bovine serum (FBS; ExCell Bio, China) at 37°C with 5% CO_2_ until reaching monolayer confluence.

Mouse peritoneal macrophages were collected as previously described (20). Briefly, 2–3 mL of 4% thioglycollate medium (Tokyo Chemical Industry Co., Ltd., Japan) was injected into the peritoneal cavity of mice. After 3–4 days, mice were anesthetized with ether, and peritoneal macrophages were collected by peritoneal lavage. The cells were suspended in RPMI 1640 medium (GIBCO, USA) supplemented with 10% FBS. Cells were seeded at a density of 1×10^6^ cells/well in 12-well plates or 2×10^5^ cells/well in 48-well plates and cultured at 37°C with 5% CO_2_ for 12 hours. Prior to each infection, cells were gently washed three times with serum-free medium. The cells were then infected with bacteria at a multiplicity of infection (MOI) of 1 for the specified duration.

### Western blotting

Following infection, cell culture supernatants were collected and concentrated using 20% (w/v) trichloroacetic acid. The cell lysates were mixed with 5× SDS loading buffer (Beyotime, China) and boiled for 10 minutes. Tissues and cell extracts were separated by 8%-12% SDS-PAGE and subsequently transferred to a polyvinylidene fluoride (PVDF) membrane via electroblotting. The membranes were blocked for 2 hours at 37°C with 5% skim milk in PBS containing 0.1% Tween-20 (PBST). Next, the PVDF membranes were incubated overnight at 4°C with primary antibodies, including anti-TLR4 (#AF8187, Beyotime, China), anti-caspase-1 (#A18646, Abclonal, China), anti-GSDMD (#ab209845, Abcam, USA), anti-Occludin (#27260-1-AP, Proteintech, China), and anti-β-actin (#52901, Signalway Antibody, USA). After washing three times with PBST, the membranes were incubated with horseradish peroxidase-labeled goat anti-mouse/rabbit IgG (#A0216/#A0277, Beyotime, China) at 37°C for 1 hour. Finally, distinct protein bands were detected using an ECL detection reagent (Beyotime, China).

### Quantitative real-time PCR analysis

Cells were prepared in 12-well plates and infected with *E. coli* as described above. Total RNA was extracted using the TRIzol reagent (Accurate Biotechnology (Human) Co., Ltd., Changsha, China). RNA concentration was measured using a spectrophotometer. The RNA was reverse transcribed using HiScript III RT SuperMix for qPCR (#R323 Vazyme, Nanning, China). Using cDNA as the template, changes in expression levels were determined by SYBR Green-based qPCR (#Q312 Vazyme, Nanning, China) using the CFX96 system (Bio-Rad, USA). The relative expression of target genes was normalized to that of GAPDH (internal reference).

### Cell death measurements

Cytotoxicity was assessed by measuring lactate dehydrogenase (LDH) release using the LDH Cytotoxicity Assay Kit (#G1780, Promega, USA) and by propidium iodide (PI) (KeyGEN Biotech, China) staining. Both the LDH measurement and PI staining were performed according to the manufacturer’s instructions.

### Immunofluorescence staining

Cell culture and infection procedures were performed as described above. After infection, cells were washed three times with PBS and fixed in 4% paraformaldehyde (Sango Biotech, Shanghai, China) for 30 minutes at room temperature (RT). Following three washes, cells were permeabilized with 0.1% Triton X-100 in PBS for 10 minutes and blocked with 5% bovine serum albumin (BSA) in PBS for 1 hour at RT. After another three washes, cells were incubated with anti-Occludin (#27260-1-AP, Proteintech, China) overnight at 4°C. The next day, cells were incubated with goat anti-rabbit IgG (H&L) Alexa Fluor 488 (Abcam, UK) for 1 hour at RT. DAPI (Beyotime, China) was added and incubated in the dark for 5 minutes. Finally, samples were mounted using anti-fade mounting medium (Solarbio, Beijing, China) and observed using fluorescence microscopy.

### ELISA

Following infection, cell culture supernatants were collected and cytokine levels were measured using enzyme-linked immunosorbent assay (ELISA) according to the manufacturer’s instructions. The ELISA kits used in this study included interleukin-1β (IL-1β), interleukin-1α (IL-1α), and interleukin-6 (IL-6) (Invitrogen, USA).

### Bacterial adhesion and invasion assays

Adhesion and invasion assays were performed as previously described with modifications (21). Briefly, after infection as described above, cells were washed three times with PBS and lysed with 300 μL of 0.1% Triton X-100 for 10 minutes at 37°C. The total bacterial count represented both adherent and invaded bacteria. For invasion assays, cells were treated with PBS containing 250 μg/mL gentamicin for 30 minutes before lysis. The number of adherent bacteria was calculated by subtracting the invasion count from the total count.

### *In vivo* infection study

TLR4^-/-^ and WT mice of similar age and weight were infected via tail vein injection with 100 μL of *E. coli* (1×10^7^ CFU), while PBS was used as a blank control. To minimize variation, mice receiving different treatments were housed in the same environment, and the order of infection and sample collection was kept consistent. Mice were monitored every 12 hours. Blood samples were collected at 4 hours post-infection for bacterial load determination. Mice were then euthanized by cervical dislocation, and brain tissues were collected for bacterial load quantification and transcriptome analysis.

### RNA sequencing and transcriptome analysis

For transcriptome analysis, brain tissues were collected and processed for total RNA extraction. Total RNA was extracted using TRIzol reagent (Invitrogen Life Technologies) and RNA quality was determined using a NanoDrop spectrophotometer (Thermo Scientific). Three micrograms of RNA were used as input material for library preparation. mRNA was purified using poly-T oligo-attached magnetic beads. First-strand cDNA was synthesized using random oligonucleotides and Super Script II, followed by second-strand synthesis using DNA Polymerase I and RNase H. After adenylation of 3’ ends, Illumina PE adapter oligonucleotides were ligated. cDNA fragments of 400–500 bp were selected using the AMPure XP system (Beckman Coulter, Beverly, CA, USA). The library was enriched using Illumina PCR Primer Cocktail in a 15-cycle PCR reaction, quantified using Agilent high sensitivity DNA assay on a Bioanalyzer 2100 system (Agilent), and sequenced on a NovaSeq 6000 platform (Illumina) by Shanghai Personal Biotechnology Co. Ltd. Raw data was filtered using fastp (v0.22.0) to obtain high-quality sequences. Filtered reads were mapped to the reference genome using HISAT2 (v2.1.0). Gene expression was quantified with HTSeq (v0.9.1) and normalized using FPKM.

Differential expression analysis was performed using DESeq2 (22). Genes with |log2FoldChange| > 1 and *p*-value < 0.05 were considered as differentially expressed genes (DEGs). Protein-protein interaction (PPI) networks were constructed using the STRING database (23). The networks were visualized using Cytoscape (24), and the top 20 hub genes showing high connectivity were identified using the Eccentricity algorithm in cytoHubba plugin (25). Functional enrichment analysis was performed using Metascape (26) and Gene Set Enrichment Analysis (GSEA) (27). Enrichment networks were visualized with terms connected by edges where similarities were above 0.3.

### Statistical analysis

Statistical analyses were conducted using GraphPad Prism 8 software (San Diego, CA). One-way analysis of variance (ANOVA) was employed to identify significant differences between groups. Results were considered statistically significant at *p < 0.05, **p < 0.01, and ***p < 0.001.

## Results

### TLR4 inhibition reduced ExPEC-induced pyroptosis in brain endothelial cells

**Figure 1:**
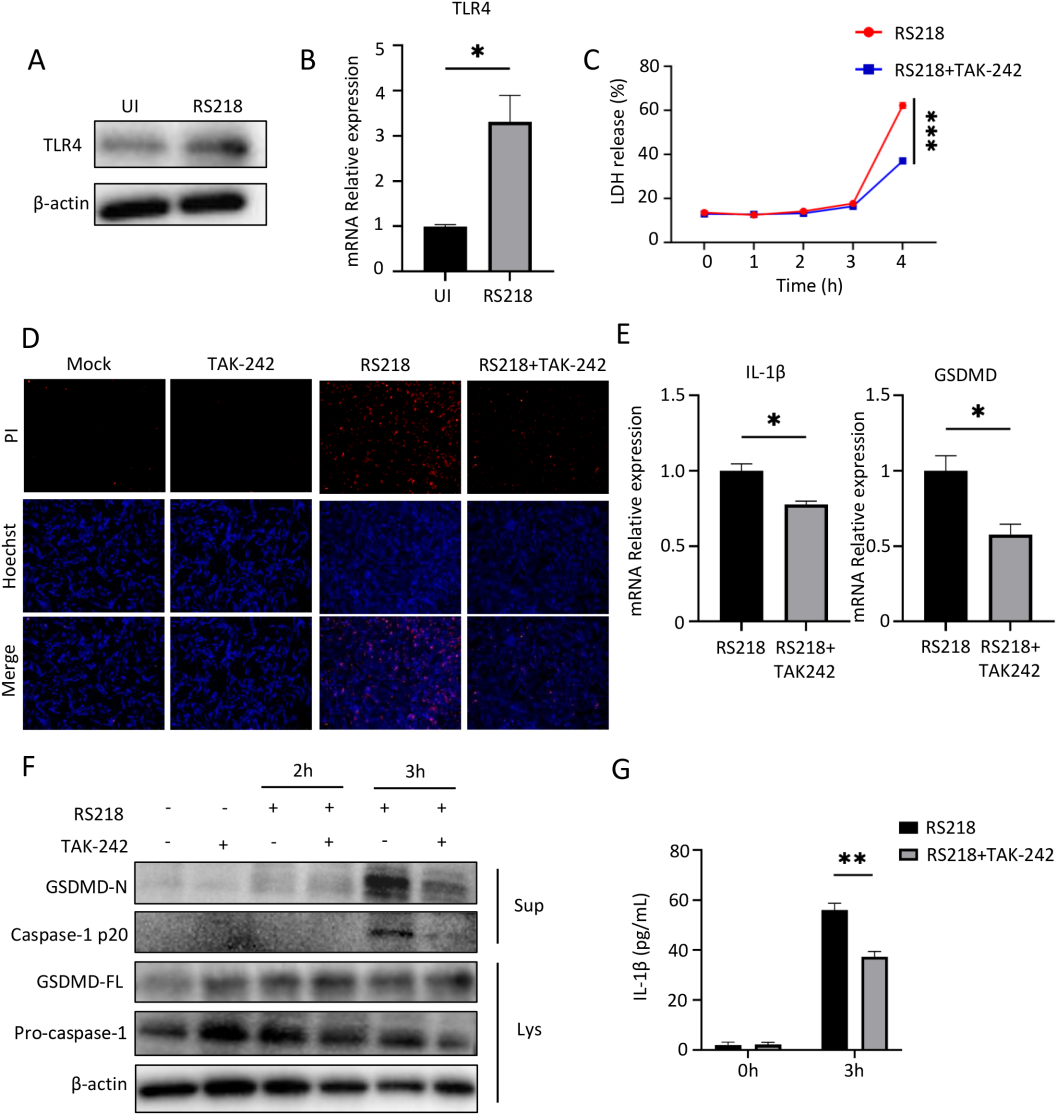
Inhibition of TLR4 reduces pyroptosis in human cerebral microvascular endothelial cells (hCMEC/D3) induced by extraintestinal pathogenic *Escherichia coli* (ExPEC). **(A)** Western blot analysis of TLR4 protein expression in hCMEC/D3 cells infected with *E coli* RS218. β-actin served as loading control. **(B)** qPCR analysis of TLR4 mRNA expression in hCMEC/D3 cells with or without RS218 infection. **(C)** Cell death was measured by LDH release assay in hCMEC/D3 cells pretreated with or without TAK-242 followed by RS218 infection for indicated time points. **(D)** Cell death visualization by PI staining (red) and Hoechst nuclear staining (blue) in hCMEC/D3 cells with indicated treatments. **(E)** qPCR analysis of IL-1β and GSDMD mRNA expression in hCMEC/D3 cells infected with RS218 with or without TAK-242 pretreatment. **(F)** Western blot analysis of pyroptosis-related proteins (GSDMD-N, Caspase-1 p20, GSDMD-FL, Pro-caspase-1) in cell lysates (Lys) and supernatants (Sup) from infected hCMEC/D3 cells. β-actin served as loading control. **(G)** ELISA analysis of IL-1β secretion in supernatants from infected hCMEC/D3 cells with indicated treatments. Data are represented as mean ± SEM. *p ≤ 0.05, **p ≤ 0.01, ***p ≤ 0.001.

To investigate the role of TLR4 in ExPEC infection, we first examined its expression in hCMEC/D3 cells infected with RS218. Western blot analysis showed increased TLR4 protein levels upon RS218 infection (**Figure 1A**), which was further supported by elevated TLR4 mRNA expression detected by qPCR (**Figure 1B**). Importantly, we observed that RS218 infection induced significant cell death in hCMEC/D3 cells (**Figures 1C, D**). Given the concurrent upregulation of TLR4, we hypothesized that TLR4 might be required for this cell death process. Indeed, pretreatment with TLR4 inhibitor TAK-242 markedly rescued RS218-induced cell death, as demonstrated by both LDH release assay (**Figure 1C**) and PI staining (**Figure 1D**).

To further characterize the type of cell death, we examined pyroptosis markers. RS218 infection triggered robust pyroptotic responses, including increased expression of IL-1β and GSDMD (**Figure 1E**), enhanced GSDMD cleavage to its active N-terminal form, activation of caspase-1 and its cleavage into the p20 subunit (**Figure 1F**), and elevated IL-1β secretion (**Figure 1G**). Notably, inhibition of TLR4 by TAK-242 significantly attenuated all these pyroptotic events. Together, these results demonstrated that TLR4 expression was upregulated during ExPEC infection, and its inhibition reduced ExPEC-induced pyroptotic cell death in brain endothelial cells.

### TLR4 inhibition enhanced ExPEC-induced tight junction disruption and bacterial invasion

**Figure 2:**
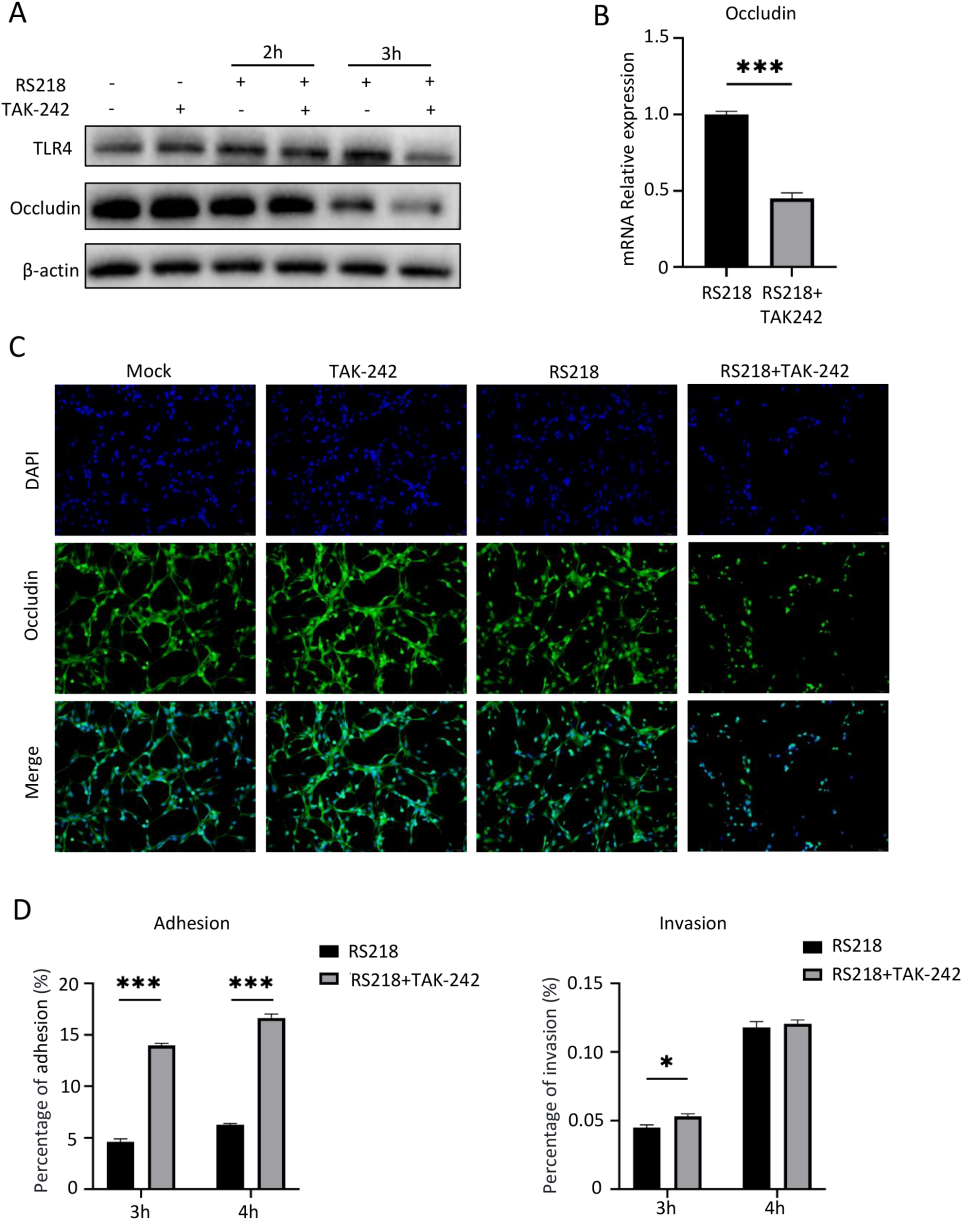
TLR4 inhibition enhances ExPEC adhesion/invasion and aggravates ExPEC-induced tight junction disruption in hCMEC/D3 cells. **(A)** Western blot analysis of TLR4 and Occludin protein expression in hCMEC/D3 cells infected with RS218 for 2h and 3h with or without TAK-242 pretreatment. β-actin served as loading control. **(B)** qPCR analysis of Occludin mRNA expression in infected hCMEC/D3 cells with indicated treatments. **(C)** Immunofluorescence analysis of Occludin expression and distribution. Nuclei were stained with DAPI (blue). **(D)** Bacterial adhesion and invasion assays in hCMEC/D3 cells at 3h and 4h post-infection with or without TAK-242 pretreatment. Data are represented as mean ± SEM. *p ≤ 0.05, ***p ≤ 0.001.

We next examined the effect of TLR4 on endothelial barrier integrity and bacterial invasion. Western blot analysis showed that RS218 infection decreased the expression of tight junction protein Occludin, and this reduction was further enhanced by TAK-242 treatment (**Figure 2A**). This pattern was further supported by decreased Occludin mRNA expression in TAK-242-treated infected cells (**Figure 2B**). Consistently, immunofluorescence microscopy revealed that RS218 infection disrupted continuous distribution of Occludin, and this disruption was more severe in the presence of TAK-242 (**Figure 2C**).

We also examined bacterial invasion under TLR4 inhibition. Bacterial adhesion assays showed that TAK-242 treatment significantly enhanced RS218 adherence to brain endothelial cells at both 3h and 4h post-infection (**Figure 2D**, left panel). Consistently, the number of intracellular bacteria was also increased in TAK-242-treated cells compared to RS218 infection alone, though this difference was only significant at 3h (**Figure 2D**, right panel). Together, these results revealed that TLR4 inhibition aggravated RS218-induced tight junction disruption and enhanced bacterial adhesion and invasion.

### TLR4 inhibition attenuated ExPEC-induced inflammatory response in macrophages

**Figure 3:**
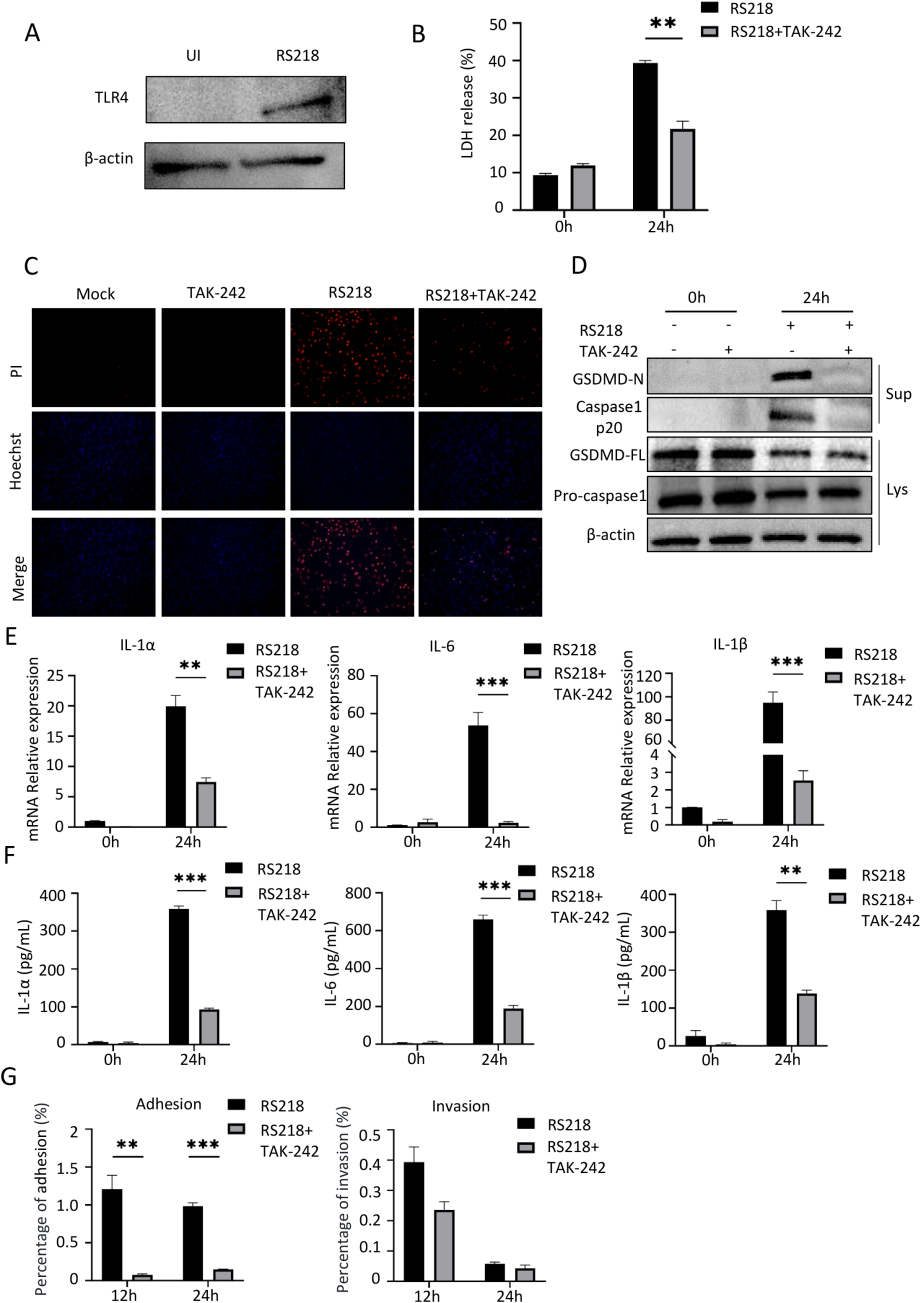
TLR4 inhibition reduces ExPEC-induced pyroptosis and inflammatory responses in mouse peritoneal macrophages. **(A)** Western blot analysis of TLR4 expression in mouse peritoneal macrophages with or without RS218 infection. **(B)** Cell death measured by LDH release in peritoneal macrophages infected with RS218 with or without TAK-242 pretreatment. **(C)** Cell death visualization by PI staining (red) and Hoechst nuclear staining (blue) in peritoneal macrophages. **(D)** Western blot analysis of pyroptosis-related proteins in cell lysates (Lys) and supernatants (Sup) from infected peritoneal macrophages with or without TAK-242 pretreatment. **(E)** mRNA expression of IL-1α, IL-6 and IL-1β in peritoneal macrophages infected with RS218 with or without TAK-242 pretreatment by qPCR. **(F)** ELISA analysis of IL-1α, IL-6 and IL-1β secretion in supernatants from peritoneal macrophages infected with RS218 with or without TAK-242 pretreatment. **(G)** Bacterial adhesion and invasion assays in peritoneal macrophages infected with RS218 with or without TAK-242 pretreatment at 12h and 24h post-infection. Data are represented as mean ± SEM. **p ≤ 0.01, ***p ≤ 0.001.

To investigate the role of TLR4 in immune cells, we used primary mouse peritoneal macrophages as a model system. Similar to endothelial cells, Western blot analysis showed that RS218 infection markedly increased TLR4 protein levels in macrophages (**Figure 3A**). RS218 infection also induced significant cell death in macrophages, and this was substantially reduced by TAK-242 treatment as demonstrated by both LDH release assay (**Figure 3B**) and PI staining (**Figure 3C**).

To examine the underlying mechanism, we analyzed pyroptosis markers in macrophages. Western blot analysis revealed that RS218 infection triggered GSDMD cleavage to its active N-terminal form and caspase-1 cleavage into the p20 subunit, while TAK-242 treatment significantly suppressed these pyroptotic events (**Figure 3D**). Similarly, RS218 infection induced robust expression of inflammatory cytokines including IL-1α, IL-6, and IL-1β, and these increases were markedly attenuated by TAK-242 treatment (**Figure 3E**). The suppression of inflammatory responses was further confirmed by reduced cytokine secretion in TAK-242-treated cells (**Figure 3F**). In contrast to endothelial cells, TAK-242 treatment significantly reduced bacterial adherence to macrophages at both 12h and 24h post-infection (**Figure 3G**, left panel). The bacterial invasion also showed a decreasing trend though not reaching statistical significance (**Figure 3G**, right panel). This distinct pattern might be attributed to the professional phagocytic nature of macrophages, where bacterial interaction is mainly mediated through specific phagocytic receptors rather than passive adhesion. Together, these results demonstrated that TLR4 inhibition reduced ExPEC-induced inflammatory responses and pyroptosis in macrophages. However, the differential effects on bacterial adhesion between these cell types highlighted the cell-type specific roles of TLR4 in host-pathogen interactions.

### TLR4 deficiency protected mice from ExPEC-induced mortality

**Figure 4:**
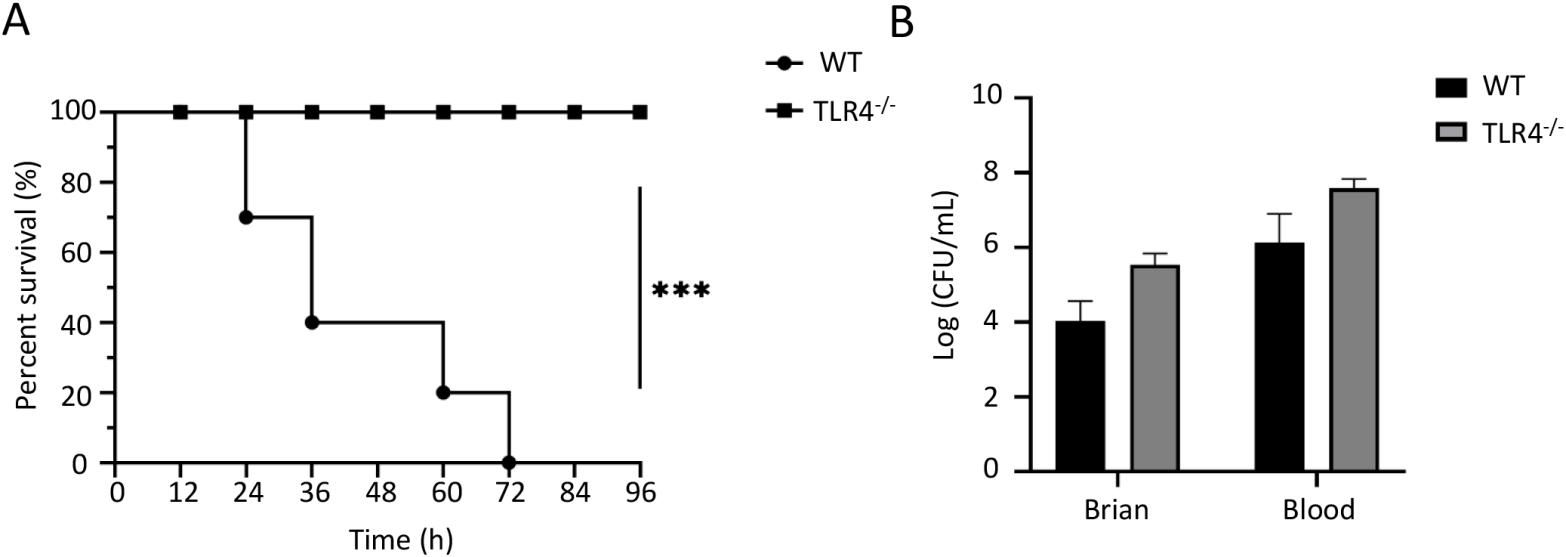
TLR4 deficiency protects mice from RS218-induced mortality but not bacterial colonization. **(A)** Survival curves of wild-type (WT) and TLR4^-/-^ mice (n=10) after intravenous infection with RS218. **(B)** Bacterial loads in brain tissue and blood from WT and TLR4^-/-^ mice (n=3) at 4h post-infection. Data are represented as mean ± SEM. ***p ≤ 0.001.

To validate our *in vitro* findings *in vivo*, we challenged wild-type (WT) and TLR4^-/-^ mice with RS218 through tail vein injection. Survival analysis showed that while all WT mice succumbed to infection within 72h, TLR4^-/-^ mice exhibited remarkable resistance with 100% survival throughout the 96h observation period (**Figure 4A**). However, when examining bacterial colonization at 4h post-infection, we found slightly higher bacterial loads in both brain tissue and blood of TLR4^-/-^ mice compared to WT mice, though the difference was not statistically significant (**Figure 4B**). This trend of increased bacterial burden might be attributed to multiple factors observed in our *in vitro* studies: enhanced bacterial adhesion/invasion and exacerbated tight junction disruption in brain endothelial cells, as well as reduced bacterial adherence in macrophages which might compromise their phagocytic clearance capacity. Nevertheless, the improved survival of TLR4^-/-^ mice despite higher bacterial loads indicated that TLR4 deficiency protected mice from ExPEC-induced mortality by modulating the host response rather than enhancing bacterial clearance, consistent with our *in vitro* observations showing the role of TLR4 in regulating inflammatory responses and cell death.

### Network analysis revealed molecular mechanisms underlying TLR4 deficiency-mediated protection

**Figure 5:**
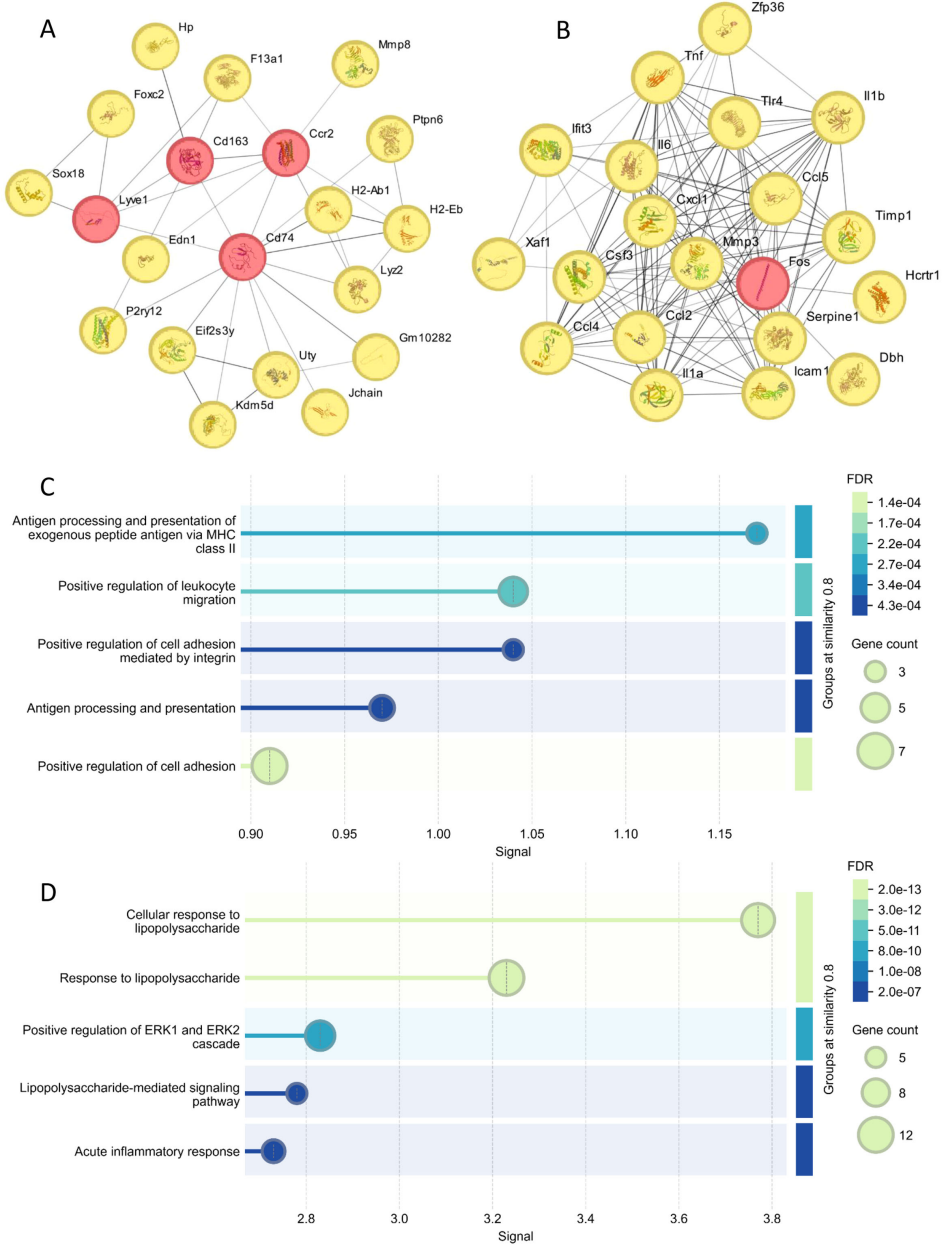
Hub gene analysis of brain transcriptome post RS218 infection in TLR4^-/-^ mice compared to wild-type mice. **(A)** Protein-protein interaction (PPI) networks were constructed using the STRING (23) database for genes upregulated in TLR4^-/-^ mice. The top 20 hub genes showing high connectivity were identified using the Eccentricity algorithm in Cytoscape (24) plugin cytoHubba (25). **(B)** PPI networks and hub gene analysis were similarly performed for downregulated genes. **(C)** Enrichment analysis of the top 20 hub genes from upregulated genes using STRING database enrichment tools. The analysis is illustrated as bubble plots with bubble size positively correlated with gene counts. **(D)** Enrichment analysis of the top 20 hub genes from downregulated genes performed and visualized using the same approach.

To understand the molecular mechanisms underlying TLR4 deficiency-mediated protection, we performed RNA sequencing analysis of brain tissues from WT and TLR4^-/-^ mice after RS218 infection. Using |log2FoldChange| > 1 and *p*-value < 0.05 as thresholds, the transcriptome analysis identified 59 upregulated genes and 205 downregulated genes in TLR4^-/-^ mice as compared to wild-type mice (**Supplementary Table S1**). Network analysis of upregulated genes in TLR4^-/-^ mice identified several important hub genes (**Figure 5A**). Among these, Cd163 is a scavenger receptor specifically expressed on monocytes/macrophages that mediates anti-inflammatory responses. Cd74, a critical component of MHC class II antigen presentation, and Lyz2, encoding lysozyme with antimicrobial activity, suggest enhanced immune defense mechanisms. These enhanced anti-inflammatory and host defense genes might explain the improved survival in TLR4^-/-^ mice despite the bacterial burden. Other upregulated hub genes included Ccr2, which is involved in leukocyte migration. Functional enrichment analysis further revealed significant enrichment of pathways related to antigen processing, immune cell migration, and cell adhesion (**Figure 5C**). The enhanced cell adhesion pathways align with our *in vitro* findings showing increased bacterial invasion and tight junction disruption in brain endothelial cells.

Analysis of downregulated genes revealed a network of inflammatory mediators as key hub genes, including pro-inflammatory cytokines (Il6, Il1a, Il1b, and Tnf), chemokines (Ccl2, Ccl4, Ccl5, and Cxcl1), and Tlr4 itself (**Figure 5B**). Notably, the reduced expression of IL-1β is consistent with our *in vitro* observations of decreased pyroptosis. These genes were significantly enriched in pathways related to cellular response to lipopolysaccharide and ERK1/2 cascade regulation (**Figure 5D**). It has been reported that ERK1/2 signaling contributes to the inflammatory response during ExPEC infection (28). The comprehensive suppression of these inflammatory pathways in TLR4^-/-^ mice provides a molecular explanation for the reduced inflammatory responses and cell death *in vitro* and improved animal survival *in vivo*.

### Functional enrichment analysis revealed distinct immune response patterns in TLR4-/- mice

**Figure 6:**
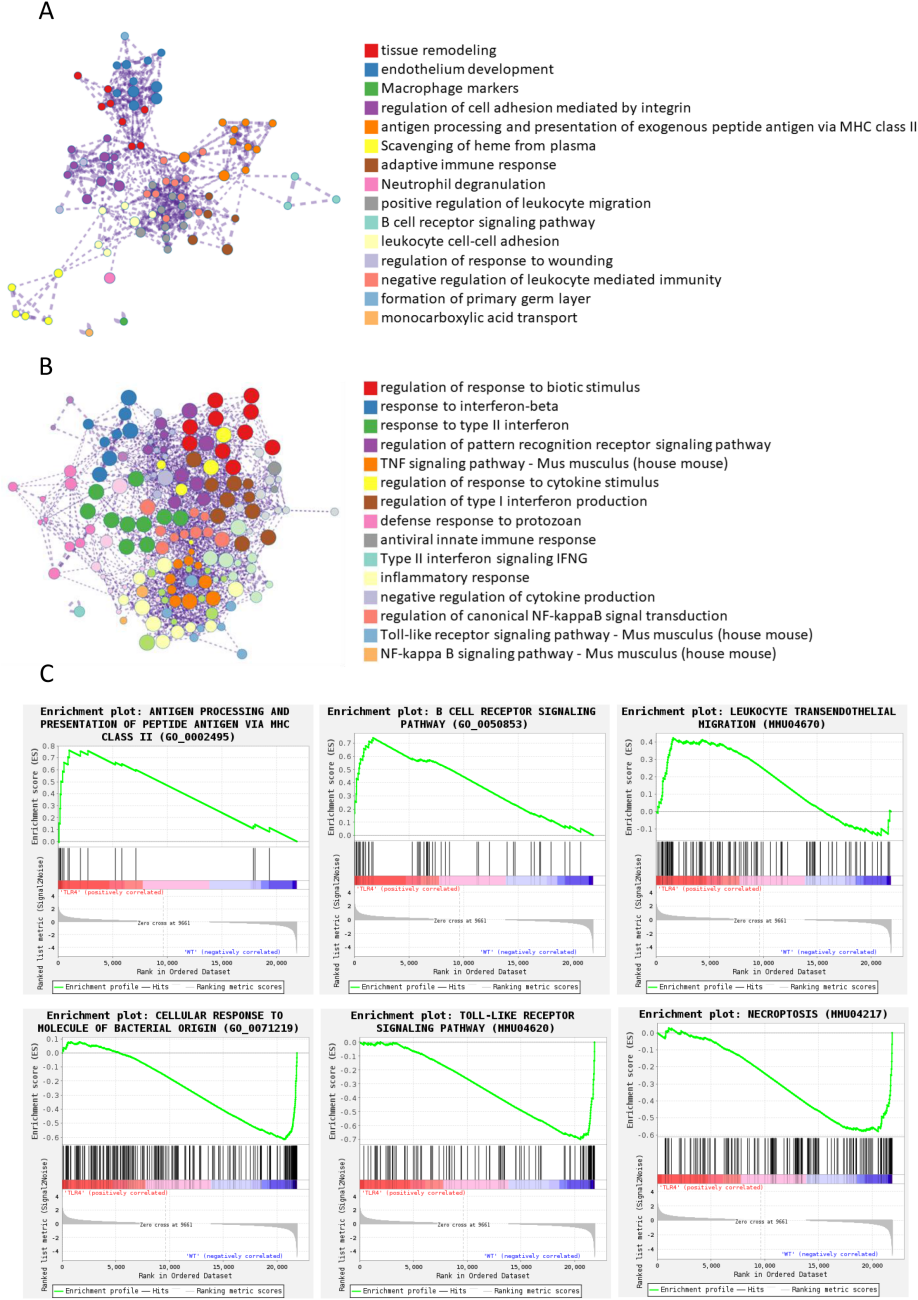
Functional enrichment analysis of differentially expressed genes between wildtype and TLR4^-/-^ mice infected with RS218. **(A)** Enrichment analysis was performed using Metascape (26) for genes upregulated in TLR4^-/-^ mice compared to wild-type mice. Networks illustrate the relationship between enriched terms, where each node represents a functional term and terms with Kappa similarities above 0.3 are connected by edges. Each term cluster is labeled by its most significant term and indicated by the same color. Different colors represent distinct functional categories as shown in the legend. **(B)** Enrichment analysis and network visualization were similarly performed for downregulated genes. **(C)** Representative pathways identified by Gene Set Enrichment Analysis (GSEA) (27) in the transcriptome analysis between wild-type and TLR4^-/-^ mice infected with RS218.

Functional enrichment analysis of upregulated genes in TLR4^-/-^ mice revealed distinct functional clusters (**Figure 6A**), including tissue remodeling, endothelium development, macrophage markers, and notably, adaptive immune responses and antigen presentation pathways. This suggested that TLR4 deficiency might trigger compensatory immune mechanisms rather than simply dampening inflammation.

In contrast, enrichment analysis of downregulated genes showed extensive suppression of pro-inflammatory pathways (**Figure 6B**), including TNF signaling, type I/II interferon responses, and pattern recognition receptor signaling. This aligned with our *in vitro* observations of reduced inflammatory cytokine production and pyroptosis in TLR4-deficient conditions. The enrichment analysis also revealed decreased toll-like receptor and NF-κB signaling pathways, confirming effective TLR4 deletion and suggesting broader suppression of inflammatory cascades.

GSEA further supported these findings, showing upregulation of antigen processing, B cell receptor signaling, and leukocyte transendothelial migration pathways in TLR4^-/-^ mice (**Figure 6C**, top panels). This coordinated upregulation of multiple immune pathways indicated that TLR4^-/-^ mice might compensate for impaired innate immunity through enhanced adaptive immune responses, providing an alternative mechanism of host defense that avoided excessive inflammatory damage. Conversely, pathways related to cellular response to bacterial molecules and toll-like receptor signaling were significantly downregulated (**Figure 6C**, bottom panels), consistent with reduced inflammatory damage observed *in vitro*. Intriguingly, the necroptosis pathway was also significantly downregulated in TLR4^-/-^ mice, suggesting that TLR4 deficiency might protect against multiple forms of cell death beyond just pyroptosis, which we observed in both endothelial cells and macrophages.

## Discussion

In this study, we uncovered a previously unrecognized role of TLR4 in regulating brain endothelial responses during ExPEC infection. While TLR4 has been traditionally viewed as a critical pattern recognition receptor for bacterial LPS detection and innate immune activation (29), our findings reveal its complex involvement in balancing protective and destructive host responses during bacterial meningitis.

Our results revealed that TLR4 deficiency triggers a comprehensive reprogramming of host response, characterized not only by reduced inflammatory damage but also enhanced tissue homeostatic processes. While pro-inflammatory pathways were suppressed, multiple protective mechanisms were upregulated, including tissue remodeling, endothelium development, heme scavenging, wound healing, and coordinated immune cell responses through enhanced antigen presentation and regulated leukocyte migration. This multi-faceted adaptation explains how TLR4^-/-^ mice survived despite higher bacterial loads: by maintaining tissue integrity and orchestrating controlled immune responses through diverse compensatory pathways. This hypothesis aligns with our *in vitro* observations: reduced pyroptosis and inflammatory cytokine production in both endothelial cells and macrophages, yet increased bacterial adhesion in endothelial cells suggesting preserved host-pathogen interactions. The transcriptome data provides molecular evidence that this phenotype results from a fundamental reprogramming of multiple cellular processes rather than a simple shift between innate and adaptive immunity. This complex adaptation allows the host to better tolerate infection while minimizing collateral tissue damage.

Previous studies have revealed several mechanisms by which meningitic *E. coli* disrupts the BBB. These include direct cellular damage caused by bacterial virulence factors (such as α-hemolysin, which affects TGFβ1-triggered hedgehog signaling) (30), host-specific factors like VEGF-A/Snail1 signaling (31), and inflammatory cytokine-mediated pathways (32). Additionally, multiple signaling molecules have been identified to regulate BBB permeability during *E. coli* meningitis, including PDGF-BB, ANGPTL4, and ARHGAP5/RhoA/MYL5 signaling cascades (32). Our current study identifies endothelial pyroptosis as a novel mechanism underlying BBB disruption during bacterial meningitis. This finding is particularly significant as it reveals how bacterial products can directly trigger programmed cell death in brain endothelial cells, leading to BBB breakdown.

Importantly, our observation aligns with the recent findings demonstrating that brain endothelial GSDMD activation mediates BBB breakdown during LPS-induced sepsis (19). While their study focused on LPS-induced BBB disruption during sepsis, our work extends this mechanism to actual bacterial infection, showing that meningitic *E. coli* can trigger GSDMD-dependent pyroptosis in brain endothelial cells. This mechanistic convergence suggests that endothelial pyroptosis may represent a common pathway through which both bacterial components (LPS) and live bacteria compromise BBB integrity. The identification of this shared mechanism not only advances our understanding of bacterial meningitis pathogenesis but also suggests potential therapeutic strategies targeting GSDMD activation in brain endothelial cells.

This discovery adds to the growing complexity of BBB regulation during bacterial meningitis and highlights how bacteria can exploit multiple pathways to breach this critical barrier. Understanding the interplay between these various mechanisms, from classical virulence factor-mediated damage to this newly identified pyroptotic pathway, will be crucial for developing more effective therapeutic strategies.

Our findings reveal that in brain endothelial cells, TLR4 inhibition enhanced bacterial adhesion and invasion while reducing pyroptotic cell death. This is consistent with previous studies showing that *E. coli* K1 strain can exploit host endothelial signaling for invasion (28). However, unlike the previously reported role of TLR2/MAPK-ERK1/2 signaling in promoting invasion, our data suggest that TLR4 may act as a restriction factor by triggering cell death and limiting bacterial entry. This differential regulation by TLR2 and TLR4 highlights the complex interplay between host receptors during infection. In contrast to our findings, the TLR2 study revealed that TLR4-deficient mice were more susceptible to meningitic *E. coli* infection. This discrepancy could be attributed to the different infection routes employed in two studies. In our intravenous infection model, bacteria directly enter the bloodstream and encounter systemic immune responses before reaching the brain, which may trigger distinct TLR4-dependent inflammatory cascades. However, in their intranasal infection model, bacteria first colonize the nasal mucosa and then potentially spread through the olfactory pathway or lymphatics to reach the brain (33, 34), which likely involves different host-pathogen interactions and immune response dynamics. These different infection routes could lead to distinct patterns of bacterial dissemination, immune cell recruitment, and inflammatory responses, ultimately resulting in different roles for TLR4 in disease progression. At the cellular level, TLR2 appears to be exploited by *E. coli* K1 to promote invasion through ERK1/2 signaling (28), while our data suggest the primary function of TLR4 may be in regulating inflammatory responses rather than directly controlling bacterial entry. This fundamental difference in TLR2 and TLR4 functions during *E. coli* meningitis provides new insights into how pathogens can differentially engage host receptors, with TLR2 being co-opted for invasion while TLR4 primarily mediates inflammatory damage.

Moreover, we found that inhibiting TLR4 further aggravated the disruption of tight junction protein during infection. This finding adds to our understanding of blood–brain barrier (BBB) regulation during bacterial meningitis. Previous studies have shown that *E. coli* can disrupt tight junctions through multiple mechanisms, including α-hemolysin-mediated effects (30) and VEGFA/Snail-1 signaling (31). Our data now suggest that TLR4-dependent inflammatory responses may actually help maintain BBB integrity, possibly through controlling excessive bacterial invasion and subsequent damage.

The unexpected improvement in survival despite increased bacterial loads and tight junction disruption in TLR4^-/-^ mice can be explained by our transcriptome analysis. The upregulation of tissue protective pathways, including enhanced endothelial development and wound healing mechanisms, suggests that TLR4 deficiency allows the host to better tolerate infection-associated damage. This aligns with recent concepts in disease tolerance, where survival of infection depends not only on pathogen control but also on the ability of host to maintain tissue integrity (35).

These findings have important therapeutic implications. Current treatments for bacterial meningitis focus primarily on bacterial elimination through antibiotics and reduction of inflammation through corticosteroids (36, 37). Our results suggest that strategies aimed at maintaining tissue homeostasis and promoting controlled immune responses, rather than complete immune suppression, might be more effective. The identification of multiple compensatory pathways in TLR4^-/-^ mice provides new targets for therapeutic intervention.

In conclusion, our study reveals TLR4 as a critical regulator of host responses during ExPEC meningitis, with its deficiency triggering complex adaptations that enhance survival through improved tissue tolerance rather than pathogen clearance. These findings provide new insights into the pathogenesis of bacterial meningitis and suggest novel therapeutic strategies focused on tissue protection and immune response modulation.

## Data Availability

The RNA sequencing data presented in the study are deposited in the NCBI Sequence Read Archive (SRA) repository under BioProject accession number PRJNA1293695, with individual sample accession numbers SAMN50031817-SAMN50031820.

## Supplementary material

Supplementary Table 1 Differentially expressed genes in brain tissue between TLR4^-/-^ and wild-type mice after RS218 infection
